# Care of the Disabled

**Published:** 1919-11-29

**Authors:** 


### CARE OF THE DISABLED.

Enham Place, the first large training-centre for partially disabled men under the auspices of the Village Centres Council, was formally opened by the Minister of Pensions on October 29. These centres are to be organised on an entirely voluntary basis with a view to assisting the State in the work of restoring incapacitated men to a position in which they can once more support themselves. They are not under the Ministry of Pensions, but the men under treatment and training receive their full pensions and allowances, which will be employed to meet the expense of their upkeep.

Enham Place is a large estate of some 1,027 acres, and is situated about three miles north of Andover Junction, on the Newbury road. On it are three hamlets and five farms, a post office, a village hall, and a club; the principal building, however, is Enham Place itself, a large country house in which accommodation is found for 100 men. It also houses the medical wing of the colony. Here are all the most recent contrivances for hydro-therapeutic treatment, such as whirlpool and running-water baths, also a complete electrical installation, for radiant heat, ionization, electrolysis, etc.

The classes of patient particularly catered for are those of the shellshock and neurasthenic types, surgical cases in the convalescent stage, and more advanced surgical cases, suffering from contractures, stiff joints, and their concomitant muscular atrophy, and cases who, as a result of severe diseases, such as malaria, etc., are obliged to seek in an outdoor life the strength which they could never find in town. Tubercular cases are not taken, they come under a separate scheme, nor are cases who can never expect to recover to a sufficient extent to earn a living taken.

The occupation advised for each resident is only chosen after mature consideration, in which the degree of disability and its nature, the educational equipment of the patient, his tastes, and general circumstances are all reviewed. There are special opportunities for agricultural and horticultural training in all their branches, including poultry, fruit and dairy farming, then those handicrafts which are invaluable in small somewhat isolated communities, as watch repairing, furniture making and mending, boot making, the repairs to agricultural machinery, the running of small power or light plants, and electric wiring, to mention but a few.

The management of the centre is under the control of Major A. Garthwaite, D.S.O., who has had much experience as a land agent; the medical arrangements are under Dr. Fortescue Fox. The residents themselves are bound by a few rules, and the aim is to attain a form of co-management, in which the men will take a large share. It is also hoped to acquire cottages in the neighbourhood where patients may instal their wives and families.

Many men will doubtless pass through this centre on their way back to their place in the ranks of the world's workers, and many who might otherwise have dragged out a miserable existence in an unsuitable occupation, and one at which they could never hope to command a full day's pay.

The organisation is supported by voluntary contributions, and though in time it may become self-supporting, at the moment there is little being earned. The medical wing, the estimated cost of which is £15,000. is to be provided by the British Red Cross Society, while the other expenses are being borne by the Village Centres Committee, of 51 Lincoln's Inn Fields.

